# Metformin directly binds the alarmin HMGB1 and inhibits its proinflammatory activity

**DOI:** 10.1074/jbc.M116.769380

**Published:** 2017-04-03

**Authors:** Takahiro Horiuchi, Natsumi Sakata, Yoshihiro Narumi, Tomohiro Kimura, Takashi Hayashi, Keisuke Nagano, Keyue Liu, Masahiro Nishibori, Sohei Tsukita, Tetsuya Yamada, Hideki Katagiri, Ryutaro Shirakawa, Hisanori Horiuchi

**Affiliations:** From the ‡ Department of Molecular and Cellular Biology, Institute of Development, Aging and Cancer, Tohoku University, 4-1 Seiryo-machi, Aoba-ku, Sendai 980-8575, Japan,; § Biomedical Technology Research Center, Tokushima Research Institute and; ¶ First Institute of New Drug Discovery, Otsuka Pharmaceutical Co., Ltd., 463–10 Kagasuno, Kawauchi-cho, Tokushima 771-0192, Japan,; ‖ Department of Pharmacology, Okayama University Graduate School of Medicine, Dentistry and Pharmaceutical Sciences, Okayama 700-8558, Japan, and; ** Department of Metabolism and Diabetes, Tohoku University Graduate School of Medicine, Sendai, 980-8575, Japan

**Keywords:** cytokine, inflammation, liver injury, metformin, p38 MAPK

## Abstract

Metformin is the first-line drug in the treatment of type 2 diabetes. In addition to its hypoglycemic effect, metformin has an anti-inflammatory function, but the precise mechanism promoting this activity remains unclear. High mobility group box 1 (HMGB1) is an alarmin that is released from necrotic cells and induces inflammatory responses by its cytokine-like activity and is, therefore, a target of anti-inflammatory therapies. Here we identified HMGB1 as a novel metformin-binding protein by affinity purification using a biotinylated metformin analogue. Metformin directly bound to the C-terminal acidic tail of HMGB1. Both *in vitro* and *in vivo*, metformin inhibited inflammatory responses induced by full-length HMGB1 but not by HMGB1 lacking the acidic tail. In an acetaminophen-induced acute liver injury model in which HMGB1 released from injured cells exacerbates the initial injury, metformin effectively reduced liver injury and had no additional inhibitory effects when the extracellular HMGB1 was blocked by anti-HMGB1-neutralizing antibody. In summary, we report for the first time that metformin suppresses inflammation by inhibiting the extracellular activity of HMGB1. Because HMGB1 plays a major role in inflammation, our results suggest possible new ways to manage HMGB1-induced inflammation.

## Introduction

Metformin, a biguanide derivative, is a hypoglycemic drug in widespread clinical use for type 2 diabetes treatment (1, 2). Metformin lowers blood glucose mainly by inhibiting gluconeogenesis in liver (1, 2). Inside cells, metformin suppresses ATP production by inhibiting mitochondrial complex 1 and glycerophosphate dehydrogenase to increase the AMP/ATP ratio that subsequently activates AMP-activated protein kinase (AMPK)^4^ (1, 2). It is proposed that the hypoglycemic effect of metformin is due to an increased AMP/ATP ratio that suppresses glucagon signaling (3) and inhibits hepatic gluconeogenic enzymes in AMPK-dependent and -independent manners (1, 2).

In addition to its hypoglycemic effect, metformin has an anti-inflammatory effect. *In vitro*, metformin suppresses lipopolysaccharide (LPS)-induced inflammatory responses in macrophages (4, 5) and endothelial cells (6). *In vivo*, metformin reduces serum levels of tumor necrosis factor α (TNFα) in high-fat diet-induced obese mice (7). At present, metformin is considered to suppress inflammation partly through improvement of blood glucose levels and more directly through AMPK-dependent and -independent inhibition of nuclear factor κB (NFκB) pathway (8).

In diabetic patients, metformin has been demonstrated to prevent various diseases such as cardiovascular diseases (9) and cancer (10, 11). Furthermore, metformin has hepatoprotective effects; it improves non-alcoholic steatohepatitis in humans (12) and prevents alcohol- and drug-induced liver injury in mice (13–16). Because inflammation is a major contributor to these diseases (17), metformin could protect these diseases at least through its anti-inflammatory function (4–6, 18, 19). However, its precise anti-inflammatory mechanism remains unclear.

Endogenous molecules released from damaged cells, termed alarmins, enhance inflammation that subsequently exacerbates the initial injury (20, 21). High mobility group box 1 (HMGB1) is a multifunctional protein acting as a DNA chaperon involved in the regulation of gene expression and also as an alarmin that extracellularly induces inflammation by stimulating variable receptors such as Toll-like receptor 4 (TLR4) and the receptor for advanced glycation end products (RAGE) (22–25). Clinically, serum HMGB1 levels are increased in patients with various inflammation-related diseases including traumatic injury, acute liver failure, myocardial infarction, and sepsis (26). Furthermore, inhibition of HMGB1 has been demonstrated to ameliorate various diseases in animal models as administration of anti-HMGB1 antibody inhibited traumatic brain injury (27) and increased survival rate of LPS-induced shock (28). These data provoke interest in HMGB1 as an attractive target for the development of new therapeutic agents for inflammation-related diseases (29–31).

In this study we identified HMGB1 as a novel metformin-binding protein and demonstrated that metformin inhibited cytokine activity of HMGB1 *in vitro* and *in vivo*. This is the first report of an extracellular function of metformin that regulates inflammation induced by HMGB1.

## Results

### Identification of HMGB1 as a metformin-binding protein

**Figure 1.**
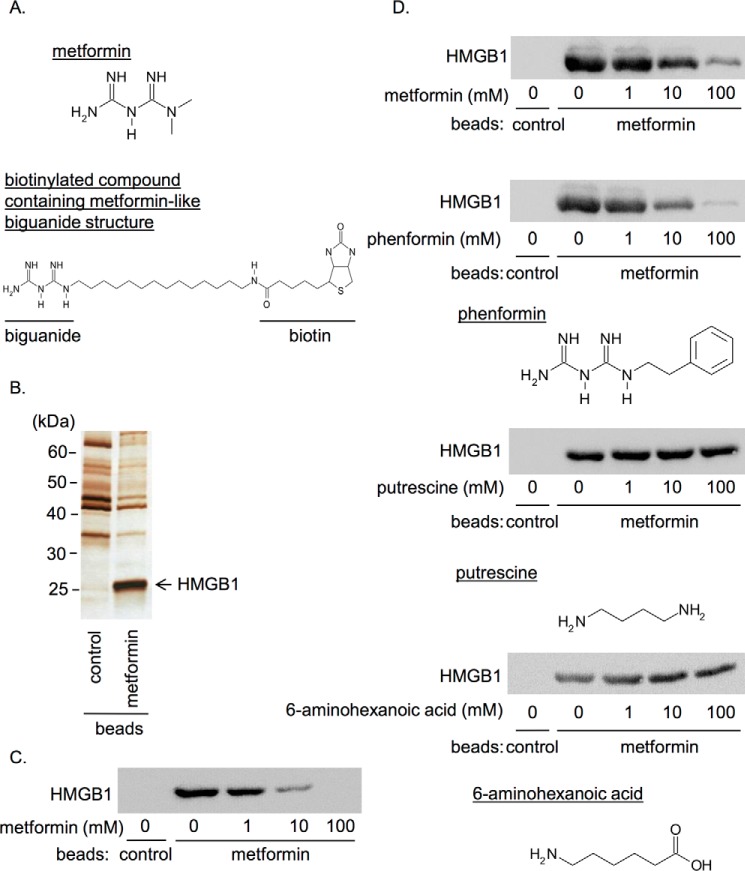
**Identification of HMGB1 as a novel metformin-binding protein.** *A*, structure of metformin (*upper*) and the biotinylated compound containing metformin-like biguanide structure (*lower*) used for the affinity chromatography. *B*, affinity purification was performed from rat liver cytosol with the metformin beads and the control biotin beads. Bead eluates were analyzed by SDS-PAGE followed by silver staining. Mass spectrometry analysis revealed the ∼25-kDa protein indicated by the *arrow* was HMGB1. *C*, HMGB1 in cytosolic fraction of HepG2 cells was pulled down with the metformin beads in the presence of indicated concentrations of metformin. *D*, recombinant HMGB1 was pulled down with the metformin beads or control beads in the presence of the indicated concentrations of metformin, phenformin, putrescine, or 6-aminohexanoic acid. Data shown are representative of three independent experiments with similar results.

To identify unknown targets of metformin, we performed affinity chromatography by using a biotinylated compound containing metformin-like biguanide structure immobilized on avidin beads (hereafter called metformin beads) (Fig. 1*A*) and rat liver cytosol. As shown in Fig. 1*B*, we specifically detected an ∼25-kDa protein in the eluate of the metformin beads but not in that of the control beads. Mass spectrometry analysis revealed the protein was HMGB1. HMGB1 in the cytosolic fraction of HepG2 hepatocellular carcinoma cells was also bound to the metformin beads, and their association was competitively inhibited by metformin in a concentration-dependent manner (Fig. 1*C*). We prepared purified recombinant HMGB1 using the baculovirus expression system. The recombinant HMGB1 bound to the metformin beads (Fig. 1*D*), indicating that their association was direct. The binding of recombinant HMGB1 to the metformin beads was concentration-dependently inhibited in the presence of metformin and also phenformin, another biguanide derivative, but not other amide-containing compounds such as putrescine and 6-aminohexanoic acid (Fig. 1*D*). Thus, the interaction of HMGB1 was specific to the biguanide structure.

### Metformin bound to the C-terminal acidic tail of HMGB1

**Figure 2.**
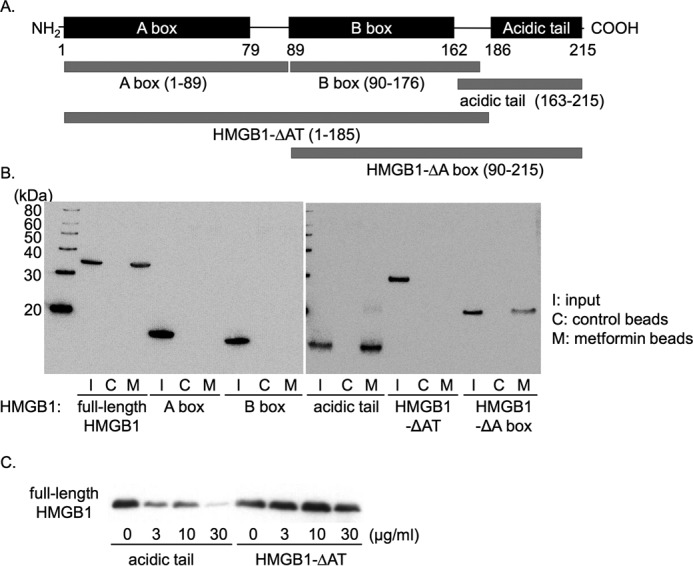
**Metformin bound to HMGB1 through its acidic tail.** *A*, domain structure of HMGB1 and its mutants used in this study. *B*, purified His-tagged HMGB1 and its mutants were pulled down with the metformin beads and detected by immunoblotting with anti-His antibody. *I*, input (30%); *C*, control beads; *M*, metformin beads. *C*, full-length HMGB1 was pulled down with the metformin beads in the presence of the indicated concentrations of the acidic tail or HMGB1-ΔAT. Data shown are representative of three independent experiments with similar results.

HMGB1 contains three major functional domains: A box domain at the N terminus, B box domain in the middle that possesses cytokine activity, and a C-terminal acidic tail domain consisting of 30 acidic amino acids (32, 33) (Fig. 2*A*). To determine the binding site of metformin, we prepared recombinant proteins of full-length HMGB1, A box, B box, an acidic tail, acidic tail-deleted HMGB1 (HMGB1-ΔAT) and A box-deleted HMGB1 (HMGB1-ΔA box) (Fig. 2*A*). Then we performed a pulldown assay with the metformin beads. As shown in Fig. 2*B*, HMGB1 mutants containing the acidic tail (full-length HMGB1, acidic tail and HMGB1-ΔA box) clearly bound to the metformin beads, whereas mutants lacking the acidic tail (A box, B box, and HMGB1-ΔAT) did not. The association between recombinant full-length HMGB1 and the metformin beads was concentration-dependently inhibited in the presence of the acidic tail but not HMGB1-ΔAT (Fig. 2*C*). These results indicated that metformin bound to the acidic tail of HMGB1.

### Metformin inhibited p38 phosphorylation induced by HMGB1 but not that induced by HMGB1-ΔAT in macrophages

**Figure 3.**
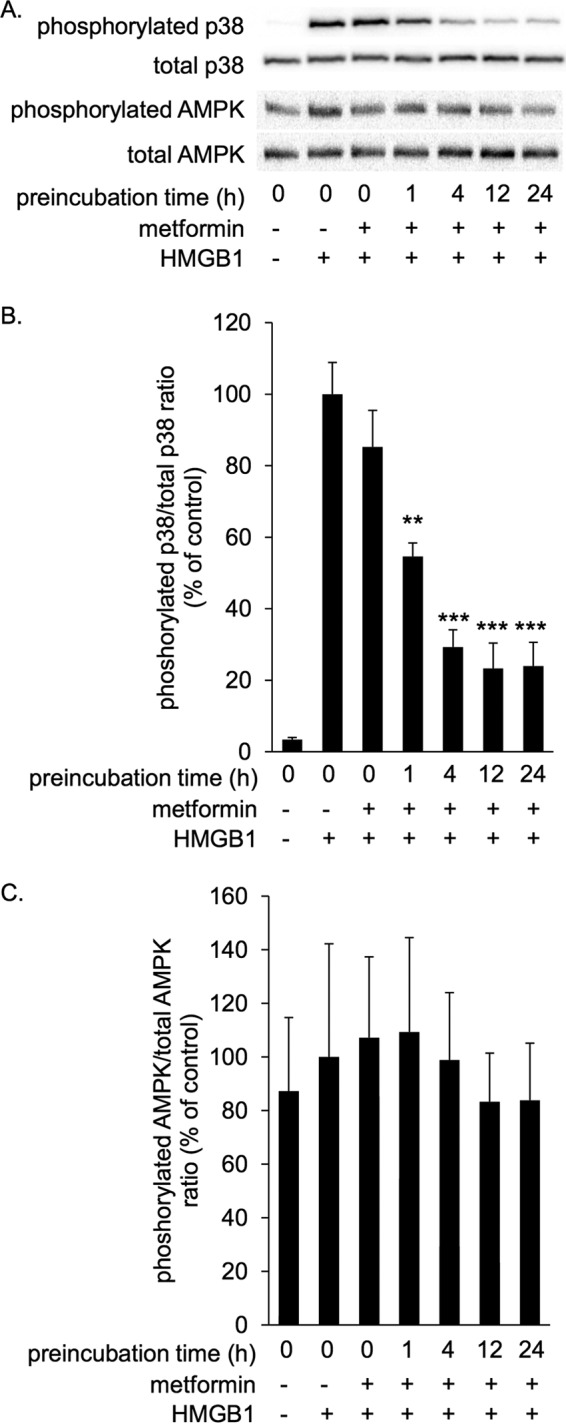
**Metformin inhibited HMGB1-induced p38 phosphorylation in RAW264.7 cells in a preincubation time-dependent manner.** RAW264.7 cells were stimulated with 1 μg/ml HMGB1 in the absence or presence of 10 mm metformin at 37 °C for 1 h. HMGB1 was preincubated with metformin at 4 °C for the indicated periods before the addition to cells. Cell lysates were subsequently analyzed by immunoblotting for phosphorylated and total p38 and phosphorylated and total AMPK. *A*, typical photos of immunoblots. Data shown are representative of four independent experiments with similar results. *B* and *C*, ratios of phosphorylated/total p38 (*B*) and phosphorylated/total AMPK (*C*) were calculated from densitometry analysis. *B* and *C*, results were analyzed as the percentage of control group (HMGB1 alone). Data are presented as the mean ± S.E. (*n* = 4). **, *p* < 0.01; ***, *p* < 0.001 *versus* control group.

HMGB1 induces proinflammatory cytokine production such as TNFα through stimulating receptors such as TLR4 and activation of p38, c-Jun N-terminal kinase (JNK), and NFκB pathway inside cells (32, 33). In the present study we focused on p38 phosphorylation as a cellular signaling response induced by HMGB1. Full-length HMGB1 or HMGB1-ΔAT at 1 μg/ml (∼40 nm) clearly induced p38 phosphorylation in RAW264.7 macrophage cells after incubation at 37 °C for 1 h (Fig. 3, *A* and *B*). This phosphorylation was strongly inhibited by a TLR4 inhibitor, TAK-242 (supplemental Fig. 1), suggesting that HMGB1 activates p38 mainly through TLR4 in RAW264.7 cells. Preincubation with metformin inhibited HMGB1-induced p38 phosphorylation (Fig. 3, *A* and *B*). It is noted that the inhibition was preincubation time-dependent (Fig. 3, *A* and *B*). In the same experiment, AMPK phosphorylation was not significantly increased by metformin (Fig. 3, *A* and *C*), probably due to short incubation periods. These results indicated that metformin inhibited the HMGB1-TLR4 pathway independently of AMPK activation.

**Figure 4.**
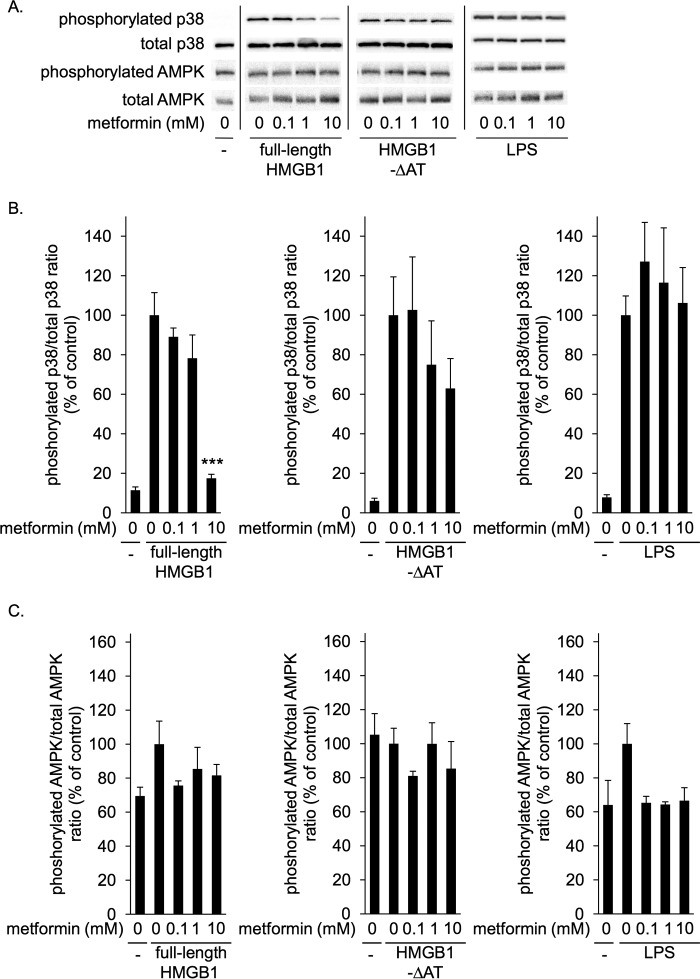
**Metformin inhibited p38 phosphorylation induced by HMGB1 but not that induced by HMGB1-ΔAT in RAW264.7 cells.** RAW264.7 cells were incubated with 1 μg/ml full-length HMGB1, HMGB1-ΔAT, or LPS in the absence or presence of indicated concentrations of metformin at 37 °C for 1 h. HMGB1 or LPS were preincubated without or with metformin at 4 °C for 24 h before the addition to cells. Cell lysates were then analyzed by immunoblotting for phosphorylated and total p38 and for phosphorylated and total AMPK. *A*, typical photos of immunoblotting. Data shown are representative of four independent experiments with similar results. *B* and *C*, ratios of phosphorylated/total p38 (*B*) and phosphorylated/total AMPK (*C*) were calculated from densitometry analysis. *B* and *C*, results were analyzed as the percentage of control group (HMGB1 alone in *left panels*, HMGB1-ΔAT alone in center panels, and LPS alone in *left panels*). Data are presented as mean ± S.E. (*n* = 4). ***; *p* < 0.001 *versus* control group.

**Figure 5.**
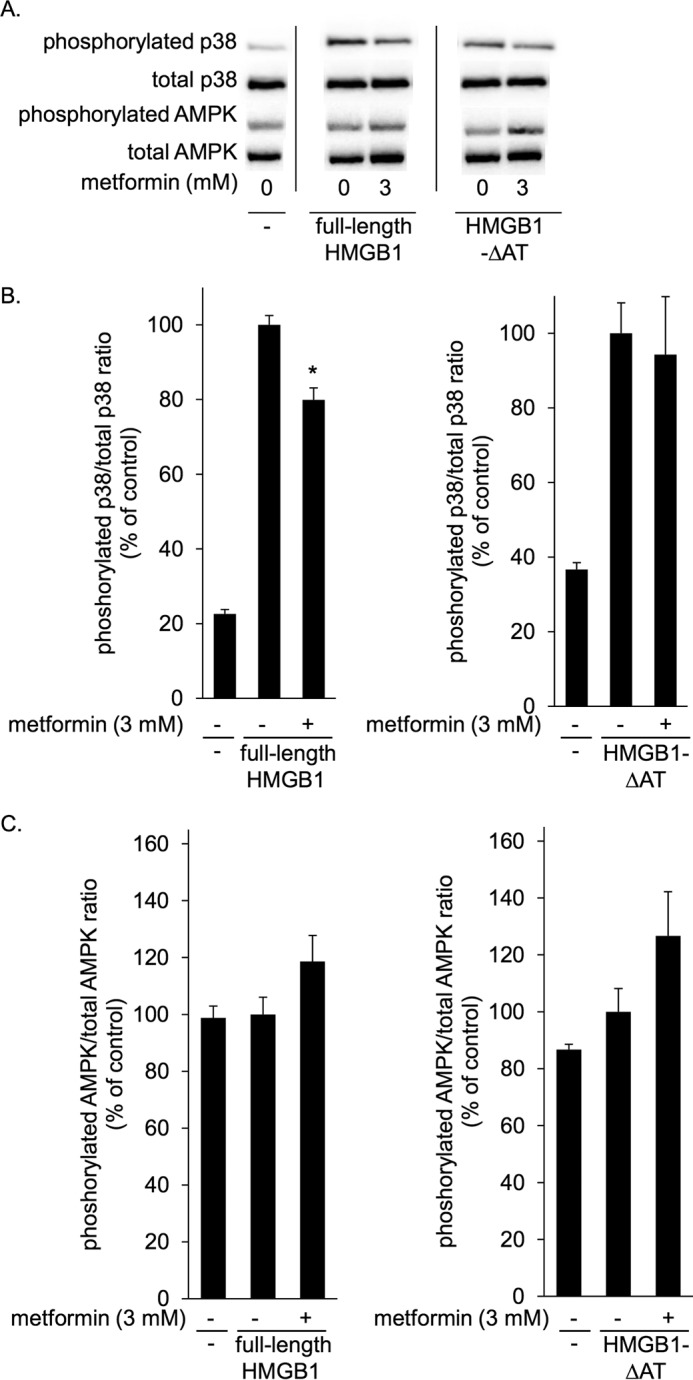
**Metformin inhibited HMGB1-induced p38 phosphorylation in mouse peritoneal macrophages.** Mouse peritoneal macrophages from Balb/c mice were incubated at 37 °C for 1 h with 1 μg/ml full-length HMGB1 or 1 μg/ml HMGB1-ΔAT in the absence or presence of 3 mm metformin. HMGB1 or HMGB1-ΔAT was preincubated without or with metformin at 4 °C for 24 h before the addition to cells. Cell lysates were then analyzed by immunoblotting for phosphorylated and total p38 and for phosphorylated and total AMPK. *A*, typical photos of immunoblots. Data shown are representative of four independent experiments with similar results. *B* and *C*, ratios of phosphorylated/total p38 (*B*) and phosphorylated/total AMPK (*C*) were calculated from densitometry analysis. *B* and *C*, results were analyzed as the percentage of control group (HMGB1 alone or HMGB1-ΔAT alone). Data are presented as mean ± S.E. (*n* = 4). *, *p* < 0.05 *versus* control group.

Metformin concentration-dependently inhibited p38 phosphorylation induced by full-length HMGB1 but not that induced by HMGB1-ΔAT (Fig. 4, *A* and *B*). Similar results were obtained with mouse peritoneal macrophages (Fig. 5, *A* and *B*). As expected, metformin did not affect p38 phosphorylation induced by LPS, a direct TLR4 agonist (Figs. 4, *A*, and *B*, and 5, *A* and *B*). Again, in these conditions metformin did not alter AMPK phosphorylation levels (Figs. 4, *A*, and *C*, and 5, *A* and *C*). Thus, metformin specifically inhibited HMGB1-induced p38 phosphorylation in RAW264.7 cells as well as mouse peritoneal macrophages through direct interaction with the acidic tail of HMGB1.

### Metformin did not alter the redox states or molecular weight of HMGB1

**Figure 6.**
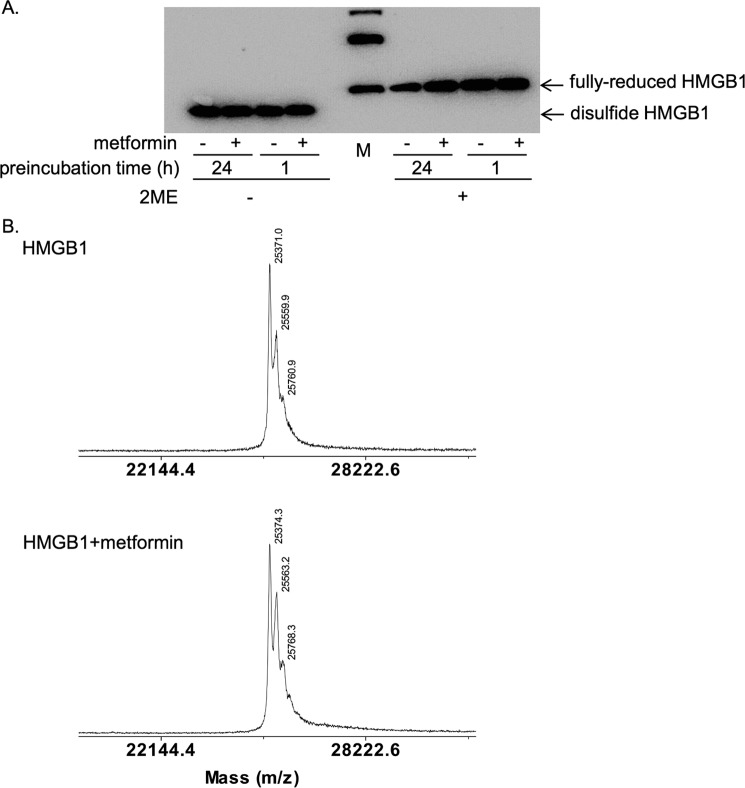
**Metformin did not change the redox states or molecular weight of HMGB1.** *A*, HMGB1 was incubated at 4 °C in the absence or presence of 100 mm metformin for the indicated time periods and analyzed by SDS-PAGE without or with β-mercaptoethanol (*2-ME*) treatment at 95 °C for 5 min. HMGB1 was detected by immunoblotting with anti-HMGB1 antibody. It is noted that disulfide and fully-reduced HMGB1 can be separated by mobility in SDS-PAGE without 2-ME treatment. Bands in the marker (*M*) lane indicated 30, 40, and 50 kDa from the bottom. *B*, mass spectrometry analysis of HMGB1 incubated without (*upper diagram*) or with 100 mm metformin (*lower diagram*) at 4 °C for 24 h.

HMGB1 contains three cysteine residues at positions 23, 45, and 106, and their redox states determine its extracellular cytokine activity (34, 35). Once released from cells, fully reduced HMGB1 is gradually oxidized to become disulfide HMGB1 with a disulfide bond between Cys-23 and Cys-45 in the A box domain (33). Disulfide HMGB1 has proinflammatory cytokine activity, whereas fully-reduced HMGB1 does not (34, 35). To clarify the molecular mechanism by which metformin inhibited HMGB1, we examined the redox states of HMGB1 by gel electrophoresis (35). The recombinant HMGB1 used in our experiments was in the disulfide form, as β-mercaptoethanol (2-ME)-treated fully-reduced HMGB1 clearly migrated slower (Fig. 6*A*) as described previously (35). After the incubation of HMGB1 with metformin at 4 °C for 1 h or 24 h, metformin did not alter the migration velocity of HMGB1, indicating that HMGB1 was not reduced by metformin (Fig. 6*A*). The data also demonstrated that HMGB1 was not degraded by incubation with metformin (Fig. 6*A*). We then examined whether metformin bound to HMGB1 with a covalent linkage such as an amide bond. Mass spectrometry analysis revealed that the molecular weight of HMGB1 was not altered after incubation with metformin at 4 °C for 24 h (Fig. 6*B*). Thus, we could not detect changes in the redox state or in the molecular weight of HMGB1 after incubation with metformin.

### Metformin inhibited TNFα elevation induced by HMGB1 but not that induced by HMGB1-ΔAT in mice

**Figure 7.**
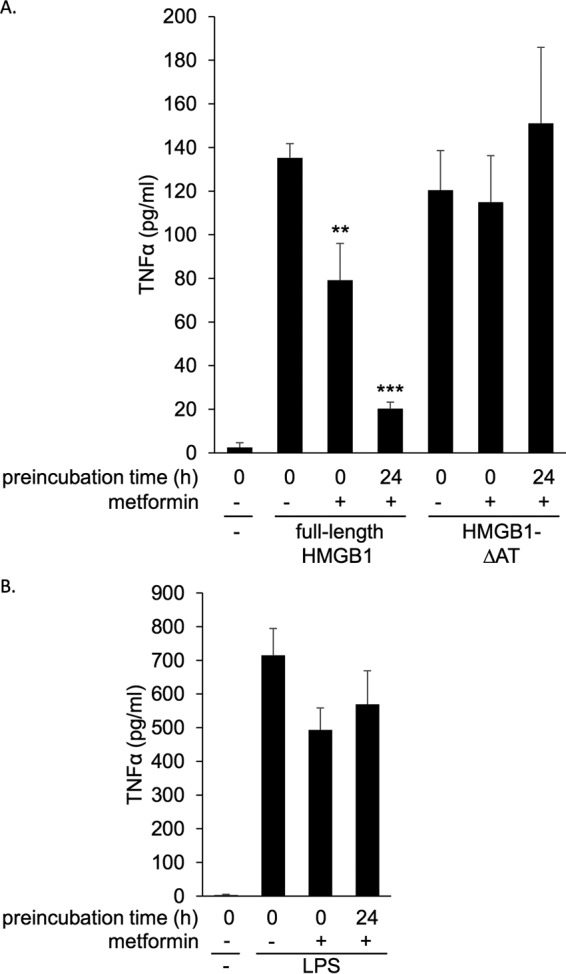
**Metformin inhibited HMGB1-induced TNFα elevation *in vivo*.** *A* and *B*, Balb/c mice were intraperitoneally administered full-length HMGB1 or HMGB1-ΔAT at 1 mg/kg (*A*) or LPS at 0.1 mg/kg (*B*) in the absence or presence of 300 mg/kg metformin. As a control, PBS vehicle was injected. In some experiments HMGB1, HMGB1-ΔAT, or LPS was preincubated with metformin at 4 °C for 24 h before injection. Serum TNFα levels were measured by ELISA 2 h after peritoneal administration. Data are presented as the mean ± S.E. (*n* = 7). **, *p* < 0.01; ***, *p* < 0.001 *versus* HMGB1 alone.

We then examined the effect of metformin on TNFα production induced by HMGB1 *in vivo* (32, 33). As shown in Fig. 7*A*, intraperitoneal administration of HMGB1 or HMGB1-ΔAT markedly increased serum TNFα levels after 2 h. Simultaneous administration of metformin inhibited HMGB1-induced elevation of TNFα levels by ∼40% but did not inhibit HMGB1-ΔAT induced elevation. When HMGB1 was preincubated with metformin at 4 °C for 24 h, the increase of TNFα levels induced by HMGB1 was more strongly inhibited, as was the case with *in vitro* experiments shown in Fig. 3. It is noted that metformin did not inhibit the HMGB1-ΔAT-induced increase of TNFα levels even after preincubation at 4 °C for 24 h. Thus, metformin inhibited HMGB1-induced elevation of TNFα *in vivo* through interaction with the acidic tail of HMGB1. As expected, metformin did not inhibit LPS-induced TNFα elevation (Fig. 7*B*), suggesting that the inhibition of TNFα elevation is specifically due to the binding of metformin with HMGB1.

### Metformin did not exert further inhibition of acetaminophen-induced liver injury in mice under blockade of HMGB1 cytokine activity

**Figure 8.**
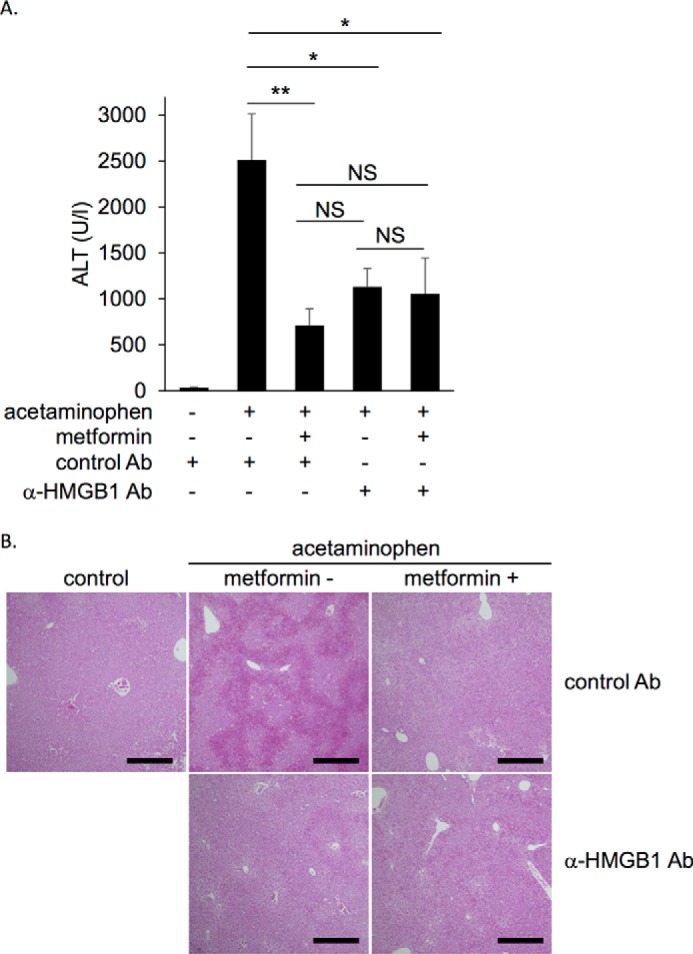
**Metformin did not exhibit further inhibition when extracellular function of HMGB1 was blocked by anti-HMGB1-neutralizing antibody in the acetaminophen-induced liver injury model mice.** Balb/c mice were intraperitoneally administered 400 mg/kg acetaminophen without or with 350 mg/kg metformin. Control antibody or anti-HMGB1 antibody at 5 mg/kg was simultaneously administered intravenously. *A*, serum ALT levels were measured 5 h after acetaminophen administration. Data are presented as the mean ± S.E. (*n* = 6–8). *, *p* < 0.05. *NS*, no significant difference. *B*, representative data of H&E staining in liver tissues 5 h after acetaminophen administration. *Scale bar*, 500 μm.

Acetaminophen overdose is well known to cause drug-induced liver injury where HMGB1 has been demonstrated to be an essential alarmin in the aggravation of the liver damage through enhancement of inflammation (36). In contrast, metformin has been demonstrated to reduce acetaminophen-induced liver injury (16). In the final sets of experiments, we examined whether metformin can inhibit endogenous HMGB1 in the acetaminophen intoxication. Metformin as well as anti-HMGB1-neutralizing monoclonal antibody efficiently suppressed the levels of alanine transaminase (ALT), a specific marker of hepatocellular damage, by ∼70% (Fig. 8*A*), and reduced the damaged area in liver parenchyma to a similar extent (Fig. 8*B*). Under the condition that extracellular HMGB1 was blocked by the anti-HMGB1-neutralizing antibody, metformin did not additionally ameliorate acetaminophen-induced liver injury and vice versa (Fig. 8). It has been demonstrated that metformin ameliorates the acetaminophen-induced liver injury by inducing gene expression of a JNK inhibiting factor, Gadd45β (16). Similar to the previous report (16), metformin showed a tendency to induce Gadd45β expression while administration of anti-HMGB1 antibody also induced it (supplemental Fig. 2). Importantly, again, this effect of anti-HMGB1 antibody was not enhanced by metformin (supplemental Fig. 2). Then, we examined whether HMGB1 targets hepatocytes using human hepatocyte cell lines. As shown in supplemental Fig. 3, recombinant HMGB1 did not induce cell injury in HepG2 hepatocytes. Although acetaminophen induced injury in the cells, the addition of recombinant HMGB1 did not increase the number of injured cells, and anti-HMGB1-neutralizing antibody did not decrease it. Furthermore, HMGB1 did not induce p38 phosphorylation in HepG2 and HuH7 hepatocytes but efficiently induced it in RAW264.7 macrophage cells under the same conditions (supplemental Fig. 4). These results suggest that the main target of HMGB1 is not hepatocytes but inflammatory cells as reported previously (36). Thus, metformin ameliorated acetaminophen-induced liver injury in mice most likely through interacting with extracellular HMGB1 and inhibiting HMGB1 cytokine activity.

## Discussion

Metformin has been demonstrated to possess an anti-inflammatory function besides its hypoglycemic effect (29–31). However, the precise mechanism of its anti-inflammatory effect remains to be clarified. In this study we identified HMGB1 as a novel metformin-binding protein (Fig. 1). We demonstrated that metformin directly bound to HMGB1 through its C-terminal acidic tail (AT) (Fig. 2) and that metformin inhibited p38 phosphorylation in macrophage cells (Figs. 4 and 5) and elevation of serum TNFα levels in mice (Fig. 7*A*) induced by full-length HMGB1 but not that induced by HMGB1-ΔAT. Furthermore, we showed that metformin most likely inhibited endogenous HMGB1 acting as an alarmin in the acetaminophen-induced liver injury mice (Fig. 8).

Metformin exerts anti-inflammatory function via AMPK-dependent and -independent mechanisms (8). Although it has been shown that metformin indirectly and slowly suppresses inflammation through activating AMPK (4, 5), the mechanism of AMPK-independent anti-inflammatory effect is not clearly elucidated. In contrast to studies that show longer exposure (about 4–24 h) to metformin activates AMPK in cultured cells (5, 37), metformin did not increase AMPK phosphorylation in our study; this is most likely due to the shorter exposure time (1 h) (Figs. 3–5). In this condition, without activation of AMPK, metformin inhibited HMGB1-induced p38 phosphorylation (Figs. 3–5). Thus, we could demonstrate that metformin inhibited the HMGB1-induced inflammatory response independently of AMPK. This is the first identification of a target of metformin that could explain AMPK-independent direct anti-inflammatory mechanism.

HMGB1 is specifically associated with the biguanide structure as metformin and another biguanide derivative, phenformin, inhibited the association of HMGB1 with the metformin beads (Fig. 1, *D* and *E*). We showed that metformin bound to the acidic tail of HMGB1 (Fig. 2*B*) but observed no changes in redox state (Fig. 6*A*) or molecular weight of HMGB1 (Fig. 6*B*) after incubation with metformin. Therefore, the molecular mechanism of metformin in the inhibition of HMGB1 cytokine activity remains elusive. The acidic tail of HMGB1, to which metformin bound, is composed exclusively of negatively charged amino acids in contrast to A box and B box containing many positively charged amino acids (26). It has been reported that the negatively charged acidic tail interacts with A box and B box domains and regulates the whole conformation of HMGB1 (38, 39). Considering biguanide is a positively charged polyamine (1, 2) (Fig. 1*A*), metformin may associate with the acidic tail through electrical interactions and may induce conformational change of whole HMGB1. Because the effective anti-HMGB1-neutralizing antibody used in this study also recognizes the acidic tail of HMGB1 (40), the acidic tail could be a reasonable target for the regulation of cytokine activity of HMGB1.

As shown in Fig. 7*A*, metformin strongly inhibited HMGB1-induced elevation of TNFα levels in mice. Similar to *in vitro* experiments, metformin did not inhibit TNFα elevation induced by HMGB1-ΔAT (Fig. 7*A*). These results indicate that the inhibitory effect of metformin in mice was indeed mediated by the direct interaction between metformin and the acidic tail of HMGB1. Importantly, this anti-inflammatory effect was observed without preincubation of HMGB1 and metformin, suggesting that metformin could interact *in vivo* with HMGB1 to inhibit its cytokine activity. Thus, the anti-inflammatory effect of metformin could be mediated by the inhibition of cytokine activity of HMGB1 *in vivo*.

Metformin is known to protect liver against injury induced by various drugs such as carbon tetrachloride (14), methotrexate (15), and acetaminophen (16). It has been demonstrated that HMGB1 released from necrotic hepatocytes plays a critical role in the acetaminophen-induced liver injury model by accelerating the primary injury (26) as the liver injury is drastically reduced in hepatocyte-specific HMGB1 knock out mice (36) or by administration of anti-HMGB1-neutralizing antibody (41). We showed that both metformin and anti-HMGB1-neutralizing antibody reduced acetaminophen-induced liver injury to the same extent and that simultaneous administration of them exhibited no additional inhibition compared with anti-HMGB1-neutralizing antibody alone (Fig. 8, supplemental Fig. 2). These results suggest that metformin acts in the same pathway with anti-HMGB1-neutralizing antibody. Given that metformin directly inhibited HMGB1 cytokine activity as demonstrated in this study, the target of metformin in this acetaminophen-induced liver injury could be endogenous HMGB1 that was released from damaged hepatocytes. In acetaminophen-induced injury in cultured hepatocytes, neither HMGB1 nor anti-HMGB1-neutralizing antibody altered the number of injured HepG2 hepatocytes. In addition, p38 phosphorylation in HepG2 and HuH7 cells was not induced by HMGB1 or LPS. This could be due to the low expression levels of TLR4 in these cells. Because it has been reported that HMGB1 enhances acetaminophen-induced liver injury by activating neutrophils (36), these results suggest that metformin reduces acetaminophen-induced liver injury via inhibition of HMGB1-induced inflammatory responses.

Metformin has been demonstrated to prevent various diseases such as cardiovascular diseases (9) and cancer (10, 11) in diabetic patients, compared with other anti-diabetic drugs under similar glycemic control levels. Interestingly, serum HMGB1 levels are shown to be significantly elevated in these diseases (42, 43). Because administration of anti-HMGB1-neutralizing antibody reduces atherosclerosis in mice (44) and a peptide that antagonizes B box cytokine activity prevents cancer progression (45–47), blockade of HMGB1 could be an effective therapeutic strategy for treating these diseases. Our results suggest that inhibition of HMGB1 could contribute to the protective effect of metformin against cardiovascular diseases and cancer in diabetic patients.

In clinical settings, the maximal approved daily dose of metformin is 2.5 g (35 mg/kg body weight) and the achievable plasma concentrations of metformin are 40–70 μm in the portal vein and 10–40 μm in the systemic circulation (48). Similar to previous studies (4, 5, 13, 16), we used 0.1–10 mm metformin for cellular experiments and 350 mg/kg for *in vivo* experiments with mice. These concentrations are apparently higher than those observed in human clinical usage. It would be important to evaluate the contribution of metformin to the inhibition of HMGB1 cytokine activity in patients treated with metformin. However, because our results showed that a high dose of metformin had an anti-inflammatory effect *in vitro* and *in vivo*, supra-therapeutic doses of metformin could potentially be useful as an anti-inflammatory drug for diseases where HMGB1 plays a critical role.

We here focused on the effect of metformin on the cytokine activity of HMGB1 that is extracellularly released. Because metformin is known to alter gene expression profiles (5, 16), metformin could affect the chromatin regulatory function of intracellular HMGB1 in cells where metformin is absorbed and concentrated. This issue remains to be clarified in future studies.

In summary, we have reported here for the first time an extracellular function of metformin that directly interacts with HMGB1 and inhibits its cytokine activity. Because HMGB1 plays a major role in the pathology of inflammation-related diseases, our results would provide a deeper understanding of the molecular action of metformin and a new perspective on the development of drugs targeting HMGB1.

## Experimental procedures

### Materials and antibodies

Metformin (1,1-dimethylbiguanide hydrochloride), phenformin (*N*-[2-phenylethyl]imidodicarbonimidic diamide monohydrochloride), and biotin were purchased from Sigma. Anti-HMGB1, anti-phosphorylated p38, anti-total p38, anti-phosphorylated AMPKα, and anti-total AMPKα antibodies were purchased from Cell Signaling Technology (Danvers, MA). Anti-His antibody was from Sigma. Horseradish peroxidase-conjugated anti-mouse IgG and anti-rabbit IgG secondary antibodies were from Jackson ImmunoResearch (West Grove, PA). Anti-HMGB1 neutralizing monoclonal antibody and control anti-keyhole limpet hemocyanin (KLH) antibody (IgG2a isotype control) were generated as described (40). LPS was from InvivoGen (San Diego, CA). TAK-242 was from EMD chemicals (San Diego, CA). Other chemicals were purchased from Sigma or Wako (Osaka, Japan).

### Identification of metformin-binding protein

All procedures were performed at 4 °C unless otherwise specified. Liver cytosol was prepared by homogenizing rat livers in buffer A (50 mm HEPES/KOH, pH 7.4, 78 mm KCl, 4 mm MgCl_2_, 2 mm EGTA, 0.2 mm CaCl_2_, 1 mm dithiothreitol) containing protease inhibitors using a glass-Teflon homogenizer. Samples were then centrifuged at 120,000 × *g* for 1 h. The supernatant was used as a rat liver cytosol. For the preparation of the affinity column, biotinylated compound containing metformin-like biguanide structure (Fig. 1*A*) or biotin as a control was immobilized on NeutrAvidin-agarose resin (Thermo Fischer Scientific, Waltham, MA). After the beads were incubated with the rat liver cytosol for 1 h, they were washed with Buffer A 4 times, and the bead-associated proteins were eluted with Buffer A containing 100 mm metformin at 30 °C for 10 min. The eluates of the metformin beads and the control beads were analyzed by sodium dodecyl sulfate-polyacrylamide gel electrophoresis (SDS-PAGE) followed by silver staining. An ∼25-kDa band observed specifically in the eluate of the metformin beads was determined by mass spectrometry analysis.

### Preparation of recombinant proteins

Human HMGB1 gene was cloned by polymerase chain reaction (PCR) amplification from human bone marrow Marathon cDNA (Clontech, Mountain View, CA). The PCR product was ligated into a pFastBacHTb vector (Invitrogen) at BamHI and XhoI sites. Mutants of HMGB1, including A box, B box, acidic tail-deleted HMGB1 (HMGB1-ΔAT), and A box-deleted HMGB1 (HMGB1-ΔA box) (Fig. 2*A*), were also cloned in a similar manner. His-tagged full-length HMGB1 and its mutants were produced in Sf9 insect cells by the baculovirus expression system. The acidic tail sequence was cloned into a pRSET A vector (Invitrogen), and the His-tagged acidic tail was produced using *Escherichia coli* strain BL21 (DE3). All His-tagged proteins were then purified on nickel nitrilotriacetic acid (NTA)-agarose beads (Qiagen, Hilden, Germany) according to the manufacturer's instructions. Full-length HMGB1 and HMGB1-ΔAT were used after cleavage of their His tags by tobacco etch virus protease (Turbo TEV protease; Accelagen, San Diego, CA) in all the experiments except for Figs. 1 and 2. All proteins were subsequently purified by gel filtration with a Superdex 200 Increase column (GE Healthcare) equilibrated with phosphate-buffered saline (PBS).

### Pulldown experiments to examine the interaction between HMGB1 and metformin

Purified recombinant HMGB1, its mutants, or the cytosolic fraction of HepG2 hepatocellular carcinoma cells was incubated at 4 °C for 1 h with the metformin beads or control biotin-immobilized beads prepared as described above in the presence of various concentrations of the competitive agents: metformin, phenformin, putrescine, 6-aminohexanoic acid (Fig. 1, *C* and *D*) as well as recombinant acidic tail or HMGB1-ΔAT (Fig. 2*C*). After the beads were washed four times, bead-associated proteins were eluted with the SDS-containing Laemmli buffer without or with β-mercaptoethanol (2-ME). They were analyzed by SDS-PAGE or by Tricine-SDS-PAGE for Fig. 2*B* followed by immunoblotting with anti-HMGB1 or anti-His antibodies and visualized by chemiluminescence reagent (Immunostar Zeta; Wako).

### Preparation of cultured cells

RAW264.7 mouse macrophage cells (RIKEN BRC Cell Bank, Ibaraki, Japan) were cultured in RPMI1640 supplemented with 10% fetal bovine serum, 100 units/ml penicillin, and 100 μg/ml streptomycin (Nacalai Tesque, Kyoto, Japan) in 5% CO_2_ at 37 °C. HepG2 and HuH7 cells were cultured in DMEM medium supplemented with 10% fetal bovine 100 units/ml penicillin and 100 μg/ml streptomycin in 5% CO_2_ at 37 °C. Mouse peritoneal macrophages were obtained by the intraperitoneal injection of 2 ml of sterile 2% thioglycollate solution (w/v) (BD Biosciences) into male Balb/c mice (8 weeks, 18–23 g; Japan SLC Inc., Hamamatsu, Japan) as described previously (49). After 4 days, peritoneal cells were collected in PBS by peritoneal lavage, and cells attached on the bottom of culture dishes after 24 h of culture were used as mouse peritoneal macrophages.

### In vitro p38 phosphorylation assay

RAW264.7 cells, mouse peritoneal macrophages, HepG2 cells, and HuH7 cells were seeded at 3 × 10^5^ cells/well in 12-well culture plate (Sumitomo Bakelite Co., Ltd., Tokyo, Japan) and cultured for 24 h. Sub-confluent cells prepared as described above were then incubated with 1 μg/ml or the indicated amounts of HMGB1, 1 μg/ml HMGB1-ΔAT, or 1 μg/ml LPS and incubated at 37 °C for 1 h. They were added to cells after preincubation without or with various concentrations of metformin at 4 °C for the indicated periods in some experiments. TAK-242 was added 30 min before adding HMGB1 at 1 μg/ml. After incubation, cells were washed with ice-cold PBS and lysed by the addition of 250 μl of buffer A containing 1% Triton X-100, 10 mm β-glycerophosphate, 10 mm sodium fluoride, 1 mm vanadate, and protease inhibitor mixture. After centrifugation at 20,000 × *g* for 10 min, supernatants of cell lysates were analyzed by SDS-PAGE followed by immunoblotting with anti-phosphorylated p38, anti-total p38, anti-phosphorylated AMPK, or anti-total AMPK antibodies. Band intensities were quantified using Image J.

### Mass spectrometry analysis

Samples were desalted using Zip Tip C4, mixed with matrix solution (10 mg/ml sinapinic acid in 0.3% TFA/70% acetonitrile), and analyzed on an AB SCIEX TOF/TOFTM5800 System (SCIEX, Framingham, MA) to determine the molecular weights.

### In vivo evaluation of HMGB1-induced TNFα levels

HMGB1 or HMGB1-ΔAT at 1 mg/kg or LPS at 0.1 mg/kg was intraperitoneally administered without or with 300 mg/kg metformin to male Balb/c mice (8 weeks old, 18–23 g; Japan SLC) after fasting for 24 h. In some experiments, HMGB1, HMGB1-ΔAT, or LPS was preincubated with metformin at 4 °C for 24 h before administration. Blood was collected after 2 h, and serum TNFα concentrations were measured by an ELISA (TNFα mouse ELISA kit; R&D systems, Minneapolis, MN) according to the manufacturer's instructions.

### Evaluation of acetaminophen-induced liver injury in a mouse model

Before acetaminophen administration, Balb/c mice (8 weeks old, 18–23 g) were fasted for 16–18 h. Acetaminophen at 400 mg/kg was intraperitoneally administered to mice without or with 350 mg/kg metformin. Anti-KLH monoclonal antibody or anti-HMGB1-neutralizing antibody at 5 mg/kg was intravenously administered to mice immediately after acetaminophen administration. Blood was collected 5 h after administration, and serum ALT concentrations were measured. At the same time, liver was fixed in 10% neutral buffered formalin (Wako), embedded in paraffin, cut into 5-μm-thick sections, and stained with hematoxylin and eosin (H&E). The sections were observed under a microscope (BZ-9000, Keyence, Osaka, Japan). Total RNA was extracted from mouse livers using an RNeasy mini kit (Qiagen). Reverse transcription was performed using ReveTra ACE qPCR RT Master Mix (TOYOBO, Osaka, Japan). Quantitative RT-PCR was performed using SYBR premix Ex TaqII (Takara, Shiga, Japan) with the CFX96 qPCR detection system (Bio-Rad). Changes of expression level of each gene were analyzed by the ΔΔCt method using mouse β-actin gene as an endogenous control. The primers for mouse Gadd45β were 5′-ATTGACATCGTCCGGGTATCAG-3′ (forward) and 5′-TTGGTTATTGCCTCTGCTCTCTT-3′ (reverse). In acetaminophen-induced cell injury assay using cultured hepatocytes, HepG2 cells were seeded at 2 × 10^4^/well in 8-well cover glass chambers (IWAKI, Sizuoka, Japan) and cultured for 20 h. Then the medium was changed to 400 μl of the medium containing 0 mm or 20 mm acetaminophen with 1.5 μg/ml anti-KLH monoclonal antibody, 1.5 μg/ml anti-HMGB1-neutralizing antibody, or 1 μg/ml HMGB1 and incubated at 37 °C for 24 h. After staining with Hoechst 33342 (DOJINDO, Kumamoto, Japan) at 1 μg/ml and propidium iodide at 0.5 μg/ml, the number of live and dead cells in randomly selected fields was visually measured using a fluorescent microscopy (BZ-9000, Keyence, Osaka, Japan).

### Statistical analysis

Data shown are expressed as the means ± S.E. Two-way analysis of variance analysis was performed. Each comparison was performed using Dunnett's test or Student's *t* test. A *p* value of <0.05 was considered significant.

### Ethical statement

This study was performed under approval of the Gene Recombination Experiment Committee and the Animal Experiment Committee of Tohoku University, Sendai, Japan.

## Author contributions

H. H. was responsible for the conception and design of the study. R. S. performed metformin affinity chromatography and designed and supervised all the experiments. T. Ho. and N. S. performed most of the experiments. Y. N. contributed to the experiment involving mouse peritoneal macrophages. T. K. supervised the experiments using molecular biological techniques. K. N. generated biotinylated compound containing metformin-like biguanide structure, and T. Ha performed the mass spectrometry analysis. K. L. and M. N. provided anti-HMGB1 and KLH antibodies. S. T., T. Y., and H. K. contributed to designing and performing the experiments of acetaminophen-induced liver injury. T. Ho., N. S., R. S., and H. H. discussed the data and drafted the paper. T. K. commented on the draft. All authors approved the final version of the manuscript.

## Supplementary Material

Supplemental Data

## Footnotes

4 The abbreviations used are:

AMPK: AMP-activated protein kinase
HMGB1: high mobility group box 1
TLR4: Toll-like receptor 4
AT: acidic tail
ALT: alanine transaminase
KLH: keyhole limpet hemocyanin
2-ME: β-mercaptoethanol.

## References

[1] Foretz M., Guigas B., Bertrand L., Pollak M., and Viollet B. (2014) Metformin: from mechanisms of action to therapies. Cell Metab. 20, 953–966 25456737 10.1016/j.cmet.2014.09.018

[2] Rena G., Pearson E. R., and Sakamoto K. (2013) Molecular mechanism of action of metformin: old or new insights? Diabetologia 56, 1898–1906 23835523 10.1007/s00125-013-2991-0 PMC3737434

[3] Miller R. A., Chu Q., Xie J., Foretz M., Viollet B., and Birnbaum M. J. (2013) Biguanides suppress hepatic glucagon signalling by decreasing production of cyclic AMP. Nature 494, 256–260 23292513 10.1038/nature11808 PMC3573218

[4] Hyun B., Shin S., Lee A., Lee S., Song Y., Ha N. J., Cho K. H., and Kim K. (2013) Metformin down-regulates TNF-α secretion via suppression of scavenger receptors in macrophages. Immune Netw. 13, 123–132 24009539 10.4110/in.2013.13.4.123 PMC3759709

[5] Kim J., Kwak H. J., Cha J. Y., Jeong Y. S., Rhee S. D., Kim K. R., and Cheon H. G. (2014) Metformin suppresses lipopolysaccharide (LPS)-induced inflammatory response in murine macrophages via activating transcription factor-3 (ATF-3) induction. J. Biol. Chem. 289, 23246–23255 24973221 10.1074/jbc.M114.577908 PMC4132821

[6] Huang N. L., Chiang S. H., Hsueh C. H., Liang Y. J., Chen Y. J., and Lai L. P. (2009) Metformin inhibits TNF-α-induced IκB kinase phosphorylation, IκB-α degradation, and IL-6 production in endothelial cells through PI3K-dependent AMPK phosphorylation. Int. J. Cardiol. 134, 169–175 18597869 10.1016/j.ijcard.2008.04.010

[7] Kim D., Lee J. E., Jung Y. J., Lee A. S., Lee S., Park S. K., Kim S. H., Park B. H., Kim W., and Kang K. P. (2013) Metformin decreases high-fat diet-induced renal injury by regulating the expression of adipokines and the renal AMP-activated protein kinase/acetyl-CoA carboxylase pathway in mice. Int. J. Mol. Med. 32, 1293–1302 24068196 10.3892/ijmm.2013.1508

[8] Saisho Y. (2015) Metformin and inflammation: its potential beyond glucose-lowering effect. Endocr. Metab. Immune Disord. Drug Targets 15, 196–205 25772174 10.2174/1871530315666150316124019

[9] UKProspective Diabetes Study (UKPDS) Group. (1998) Effect of intensive blood-glucose control with metformin on complications in overweight patients with type 2 diabetes (UKPDS 34). Lancet 352, 854–865 9742977

[10] Gronich N., and Rennert G. (2013) Beyond aspirin-cancer prevention with statins, metformin, and bisphosphonates. Nat. Rev. Clin. Oncol. 10, 625–642 24080598 10.1038/nrclinonc.2013.169

[11] Gandini S., Puntoni M., Heckman-Stoddard B. M., Dunn B. K., Ford L., DeCensi A., and Szabo E. (2014) Metformin and cancer risk and mortality: a systematic review and meta-analysis taking into account biases and confounders. Cancer Prev. Res. 7, 867–885 10.1158/1940-6207.CAPR-13-0424 PMC4154969 24985407

[12] Marchesini G., Brizi M., Bianchi G., Tomassetti S., Zoli M., and Melchionda N. (2001) Metformin in non-alcoholic steatohepatitis. Lancet 358, 893–894 11567710 10.1016/s0140-6736(01)06042-1

[13] Bergheim I., Guo L., Davis M. A., Lambert J. C., Beier J. I., Duveau I., Luyendyk J. P., Roth R. A., and Arteel G. E. (2006) Metformin prevents alcohol-induced liver injury in the mouse: critical role of plasminogen activator inhibitor-1. Gastroenterology 130, 2099–2112 16762632 10.1053/j.gastro.2006.03.020 PMC2648856

[14] Poon M. K., Chiu P. Y., Mak D. H., and Ko K. M. (2003) Metformin protects against carbon tetrachloride hepatotoxicity in mice. J. Pharmacol. Sci. 93, 501–504 14737024 10.1254/jphs.93.501

[15] Hadi N. R., Al-Amran F. G., and Swadi A. (2012) Metformin ameliorates methotrexate-induced hepatotoxicity. J. Pharmacol. Pharmacother. 3, 248–253 23129960 10.4103/0976-500X.99426 PMC3487273

[16] Kim Y. H., Hwang J. H., Kim K. S., Noh J. R., Choi D. H., Kim D. K., Tadi S., Yim Y. H., Choi H. S., and Lee C. H. (2015) Metformin ameliorates acetaminophen hepatotoxicity via Gadd45β-dependent regulation of JNK signaling in mice. J. Hepatol. 63, 75–82 25681557 10.1016/j.jhep.2015.02.008

[17] Chawla A., Nguyen K. D., and Goh Y. P. (2011) Macrophage-mediated inflammation in metabolic disease. Nat. Rev. Immunol. 11, 738–749 21984069 10.1038/nri3071 PMC3383854

[18] Hattori Y., Hattori K., and Hayashi T. (2015) Pleiotropic benefits of metformin: macrophage targeting its anti-inflammatory mechanisms. Diabetes 64, 1907–1909 25999535 10.2337/db15-0090

[19] Scheen A. J., Esser N., and Paquot N. (2015) Antidiabetic agents: potential anti-inflammatory activity beyond glucose control. Diabetes Metab. 41, 183–194 25794703 10.1016/j.diabet.2015.02.003

[20] Kono H., and Rock K. L. (2008) How dying cells alert the immune system to danger. Nat. Rev. Immunol. 8, 279–289 18340345 10.1038/nri2215 PMC2763408

[21] Lotze M. T., Deisseroth A., and Rubartelli A. (2007) Damage associated molecular pattern molecules. Clin. Immunol. 124, 1–4 17468050 10.1016/j.clim.2007.02.006 PMC2000827

[22] Lotze M. T., and Tracey K. J. (2005) High-mobility group box 1 protein (HMGB1): nuclear weapon in the immune arsenal. Nat. Rev. Immunol. 5, 331–342 15803152 10.1038/nri1594

[23] Müller S., Scaffidi P., Degryse B., Bonaldi T., Ronfani L., Agresti A., Beltrame M., and Bianchi M. E. (2001) New EMBO members' review: the double life of HMGB1 chromatin protein: architectural factor and extracellular signal. EMBO J. 20, 4337–4340 11500360 10.1093/emboj/20.16.4337 PMC125571

[24] Bianchi M. E., Falciola L., Ferrari S., and Lilley D. M. (1992) The DNA binding site of HMG1 protein is composed of two similar segments (HMG boxes), both of which have counterparts in other eukaryotic regulatory proteins. EMBO J. 11, 1055–1063 1547772 10.1002/j.1460-2075.1992.tb05144.x PMC556546

[25] Rubartelli A., and Lotze M. T. (2007) Inside, outside, upside down: damage-associated molecular-pattern molecules (DAMPs) and redox. Trends Immunol. 28, 429–436 17845865 10.1016/j.it.2007.08.004

[26] Kang R., Chen R., Zhang Q., Hou W., Wu S., Cao L., Huang J., Yu Y., Fan X. G., Yan Z., Sun X., Wang H., Wang Q., Tsung A., Billiar T. R., et al (2014) HMGB1 in health and disease. Mol. Aspects Med. 40, 1–116 25010388 10.1016/j.mam.2014.05.001 PMC4254084

[27] Okuma Y., Liu K., Wake H., Zhang J., Maruo T., Date I., Yoshino T., Ohtsuka A., Otani N., Tomura S., Shima K., Yamamoto Y., Yamamoto H., Takahashi H. K., Mori S., and Nishibori M. (2012) Anti-high mobility group box-1 antibody therapy for traumatic brain injury. Ann. Neurol. 72, 373–384 22915134 10.1002/ana.23602

[28] Wang H., Bloom O., Zhang M., Vishnubhakat J. M., Ombrellino M., Che J., Frazier A., Yang H., Ivanova S., Borovikova L., Manogue K. R., Faist E., Abraham E., Andersson J., Andersson U., et al (1999) HMG-1 as a late mediator of endotoxin lethality in mice. Science 285, 248–251 10398600 10.1126/science.285.5425.248

[29] Yang H., Ochani M., Li J., Qiang X., Tanovic M., Harris H. E., Susarla S. M., Ulloa L., Wang H., DiRaimo R., Czura C. J., Wang H., Roth J., Warren H. S., Fink M. P., et al (2004) Reversing established sepsis with antagonists of endogenous high-mobility group box 1. Proc. Natl. Acad. Sci. U.S.A. 101, 296–301 14695889 10.1073/pnas.2434651100 PMC314179

[30] Fink M. P. (2007) Bench-to-bedside review: High-mobility group box 1 and critical illness. Crit. Care 11, 229 17903310 10.1186/cc6088 PMC2556731

[31] Andersson U., and Tracey K. J. (2011) HMGB1 is a therapeutic target for sterile inflammation and infection. Annu. Rev. Immunol. 29, 139–162 21219181 10.1146/annurev-immunol-030409-101323 PMC4536551

[32] Huang W., Tang Y., and Li L. (2010) HMGB1, a potent proinflammatory cytokine in sepsis. Cytokine 51, 119–126 20347329 10.1016/j.cyto.2010.02.021

[33] Tang D., Kang R., Zeh H. J. 3rd, and Lotze M. T. (2011) High-mobility group box 1, oxidative stress, and disease. Antioxid. Redox Signal. 14, 1315–1335 20969478 10.1089/ars.2010.3356 PMC3048826

[34] Yang H., Lundbäck P., Ottosson L., Erlandsson-Harris H., Venereau E., Bianchi M. E., Al-Abed Y., Andersson U., Tracey K. J., and Antoine D. J. (2012) Redox modification of cysteine residues regulates the cytokine activity of high mobility group box-1 (HMGB1). Mol. Med. 18, 250–259 22105604 10.2119/molmed.2011.00389 PMC3324950

[35] Venereau E., Casalgrandi M., Schiraldi M., Antoine D. J., Cattaneo A., De Marchis F., Liu J., Antonelli A., Preti A., Raeli L., Shams S. S., Yang H., Varani L., Andersson U., Tracey K. J., et al (2012) Mutually exclusive redox forms of HMGB1 promote cell recruitment or proinflammatory cytokine release. J. Exp. Med. 209, 1519–1528 22869893 10.1084/jem.20120189 PMC3428943

[36] Huebener P., Pradere J. P., Hernandez C., Gwak G. Y., Caviglia J. M., Mu X., Loike J. D., Jenkins R. E., Antoine D. J., and Schwabe R. F. (2015) The HMGB1/RAGE axis triggers neutrophil-mediated injury amplification following necrosis. J. Clin. Invest. 125, 539–550 25562324 10.1172/JCI76887 PMC4319429

[37] Tsoyi K., Jang H. J., Nizamutdinova I. T., Kim Y. M., Lee Y. S., Kim H. J., Seo H. G., Lee J. H., and Chang K. C. (2011) Metformin inhibits HMGB1 release in LPS-treated RAW 264.7 cells and increases survival rate of endotoxaemic mice. Br. J. Pharmacol. 162, 1498–1508 21091653 10.1111/j.1476-5381.2010.01126.x PMC3057288

[38] Ramstein J., Locker D., Bianchi M. E., and Leng M. (1999) Domain-domain interactions in high mobility group 1 protein (HMG1). Eur. J. Biochem 260, 692–700 10102997 10.1046/j.1432-1327.1999.00185.x

[39] Stott K., Watson M., Howe F. S., Grossmann J. G., and Thomas J. O. (2010) Tail-mediated collapse of HMGB1 is dynamic and occurs via differential binding of the acidic tail to the A and B domains. J. Mol. Biol. 403, 706–722 20691192 10.1016/j.jmb.2010.07.045

[40] Liu K., Mori S., Takahashi H. K., Tomono Y., Wake H., Kanke T., Sato Y., Hiraga N., Adachi N., Yoshino T., and Nishibori M. (2007) Anti-high mobility group box 1 monoclonal antibody ameliorates brain infarction induced by transient ischemia in rats. FASEB J. 21, 3904–3916 17628015 10.1096/fj.07-8770com

[41] Yang R., Zou X., Tenhunen J., Zhu S., Kajander H., Koskinen M. L., and Tonnessen T. I. (2014) HMGB1 neutralization is associated with bacterial translocation during acetaminophen hepatotoxicity. BMC Gastroenterol. 14, 66 24708589 10.1186/1471-230X-14-66 PMC3985724

[42] Goldstein R. S., Gallowitsch-Puerta M., Yang L., Rosas-Ballina M., Huston J. M., Czura C. J., Lee D. C., Ward M. F., Bruchfeld A. N., Wang H., Lesser M. L., Church A. L., Litroff A. H., Sama A. E., and Tracey K. J. (2006) Elevated high-mobility group box 1 levels in patients with cerebral and myocardial ischemia. Shock 25, 571–574 16721263 10.1097/01.shk.0000209540.99176.72

[43] Tang D., Kang R., Zeh H. J. 3rd, and Lotze M. T. (2010) High-mobility group box 1 and cancer. Biochim. Biophys. Acta 1799, 131–140 20123075 10.1016/j.bbagrm.2009.11.014 PMC2818552

[44] Kanellakis P., Agrotis A., Kyaw T. S., Koulis C., Ahrens I., Mori S., Takahashi H. K., Liu K., Peter K., Nishibori M., and Bobik A. (2011) High-mobility group box protein 1 neutralization reduces development of diet-induced atherosclerosis in apolipoprotein e-deficient mice. Arterioscler. Thromb. Vasc. Biol. 31, 313–319 21088249 10.1161/ATVBAHA.110.218669

[45] Agresti A., Lupo R., Bianchi M. E., and Müller S. (2003) HMGB1 interacts differentially with members of the Rel family of transcription factors. Biochem. Biophys. Res. Commun. 302, 421–426 12604365 10.1016/s0006-291x(03)00184-0

[46] Lotze M. T., and DeMarco R. A. (2003) Dealing with death: HMGB1 as a novel target for cancer therapy. Curr. Opin. Investig. Drugs 4, 1405–1409 14763124

[47] Ellerman J. E., Brown C. K., de Vera M., Zeh H. J., Billiar T., Rubartelli A., and Lotze M. T. (2007) Masquerader: high mobility group box-1 and cancer. Clin. Cancer Res. 13, 2836–2848 17504981 10.1158/1078-0432.CCR-06-1953

[48] He L., and Wondisford F. E. (2015) Metformin action: concentrations matter. Cell Metab. 21, 159–162 25651170 10.1016/j.cmet.2015.01.003

[49] Li J., Kokkola R., Tabibzadeh S., Yang R., Ochani M., Qiang X., Harris H. E., Czura C. J., Wang H., Ulloa L., Wang H., Warren H. S., Moldawer L. L., Fink M. P., Andersson U., Tracey K. J., and Yang H. (2003) Structural basis for the proinflammatory cytokine activity of high mobility group box 1. Mol. Med. 9, 37–45 12765338 PMC1430376

