# Supplementary material for: Metformin directly binds the alarmin HMGB1 and inhibits its proinflammatory activity
Source: J Biol Chem. 2017 Apr 3;292(20):8436–46. doi: 10.1074/jbc.M116.769380 (PMC5437248; doi:10.1074/jbc.M116.769380)
Supplement: Supplemental Data [file supp_292_20_8436__index.html]

Metformin Directly Binds the Alarmin HMGB1 and Inhibits its Proinflammatory Activity — Metformin directly binds the alarmin HMGB1 and inhibits its proinflammatory activity — Metformin Inhibits HMGB1 Proinflammatory Cytokine Activity — Supplemental Data 

# Metformin directly binds the alarmin HMGB1 and inhibits its proinflammatory activity

## Supplemental Data

- Supplemental Figure. 1-4 (.pdf, 446 KB) - Supplemental Figure 1. TLR4 inhibitor reduced HMGB1-induced p38 phosphorylation in RAW 264.7 cells Supplemental Figure 2. Metformin and anti-HMGB1 antibody induce change of gene expression in acetaminophen-induced liver injury model Supplemental Figure. 3 HMGB1 had no effect on the acetaminophen-induced injury of hepatocytes in vitro Supplemental Figure. 4: HMGB1 induced no inflammatory response in cultured hepatocytes
