# Supplementary material for: Metformin directly binds the alarmin HMGB1 and inhibits its proinflammatory activity
Source: J Biol Chem. 2017 Apr 3;292(20):8436–46. doi: 10.1074/jbc.M116.769380 (PMC5437248; doi:10.1074/jbc.M116.769380)
Supplement: Supplemental Data [file 10.1074_M116.769380_jbc.M116.769380-1.pdf]

Supplemental data

Metformin Directly Binds the Alarmin HMGB1 and Inhibits its Proinflammatory Activity

Takahiro Horiuchi[1†], Natsumi Sakata[1†], Yoshihiro Narumi[1], Tomohiro Kimura[1], Takashi Hayashi[2], Keisuke Nagano[3], Keyue Liu[4], Masahiro Nishibori[4], Sohei Tsukita[5], Tetsuya Yamada[5], Hideki Katagiri[5], Ryutaro Shirakawa[1*], and Hisanori Horiuchi[1*]

[1]Department of Molecular and Cellular Biology, Institute of Development, Aging and Cancer, Tohoku University, 4-1 Seiryo-machi, Aoba-ku, Sendai 980-8575, Japan [2]Biomedical, Technology Research Center, and [3]First Institute of New Drug Discovery, Tokushima Research Institute, Otsuka Pharmaceutical Co., Ltd., 463-10 Kagasuno, Kawauchi-cho, Tokushima 771-0192, Japan [4]Department of Pharmacology, Okayama University Graduate School of Medicine, Dentistry and Pharmaceutical Sciences, Okayama 700-8558, Japan [5]Department of Metabolism and Diabetes, Tohoku University Graduate School of Medicine, Sendai, 980-8575, Japan

Running title: Metformin Inhibits HMGB1 Proinflammatory Cytokine Activity

*; To whom correspondence should be addressed: R.S.: tel. +81-22-717-8575, fax. +81-22-717-8463, E-mail; ryutaro.shirakawa.b1@tohoku.ac.jp; H.H.: tel. & fax. +81-22-717-8463, E-mail; hisanori.horiuchi.e8@tohoku.ac.jp
†; Co-first author

Table of Contents

- Supplemental Figure. 1
- Supplemental Figure. 2
- Supplemental Figure. 3
- Supplemental Figure. 4

Supplemental Figure. 1

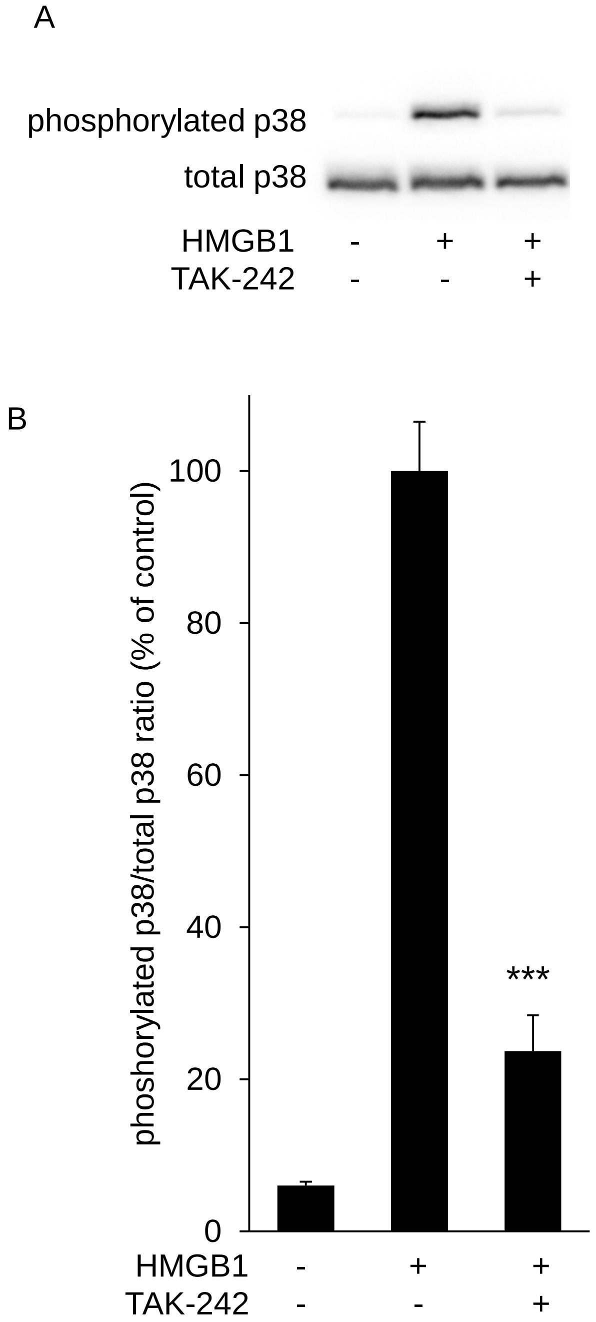

Supplemental Figure 1. TLR4 inhibitor reduced HMGB1-induced p38 phosphorylation in RAW 264.7 cells

RAW264.7 cells were treated with 1 µg/ml TLR4 inhibitor TAK-242 at 37℃ for 30min and then stimulated with 1 µg/ml HMGB1 at 37 ℃ for 1 h. Cell lysates were subsequently analyzed by immunoblotting for phosphorylated and total p38. (A) Typical photos of immunoblots. Data shown are representative of four independent experiments with similar results. (B) Ratios of phosphorylated/total p38 were calculated from densitometry analysis. Results were analyzed as the percentage of control group (HMGB1 alone). Data are presented as mean ± SEM (n=4). ***; *P* < 0.001 vs. control group.

Supplemental Figure. 2

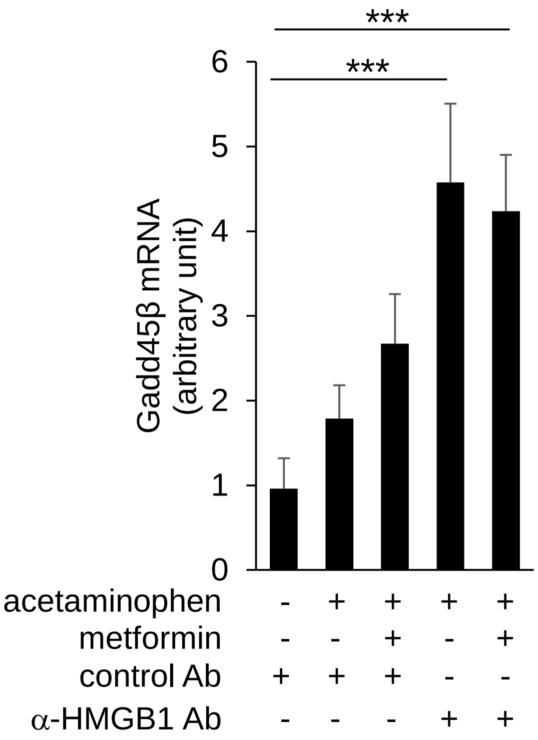

Supplemental Figure 2. Metformin and anti-HMGB1 antibody induce change of gene expression in acetaminophen-induced liver injury model
Balb/c mice were intraperitoneally administered 400 mg/kg acetaminophen without or with 350 mg/kg metformin. Control antibody or anti-HMGB1 antibody at 5 mg/kg was simultaneously administered intravenously. Relative expression levels of Gadd45β after 5h in liver were measured. Data are presented as mean ± SEM (n=6-8). ***; $P<0.01$.

Supplemental Figure. 3

A

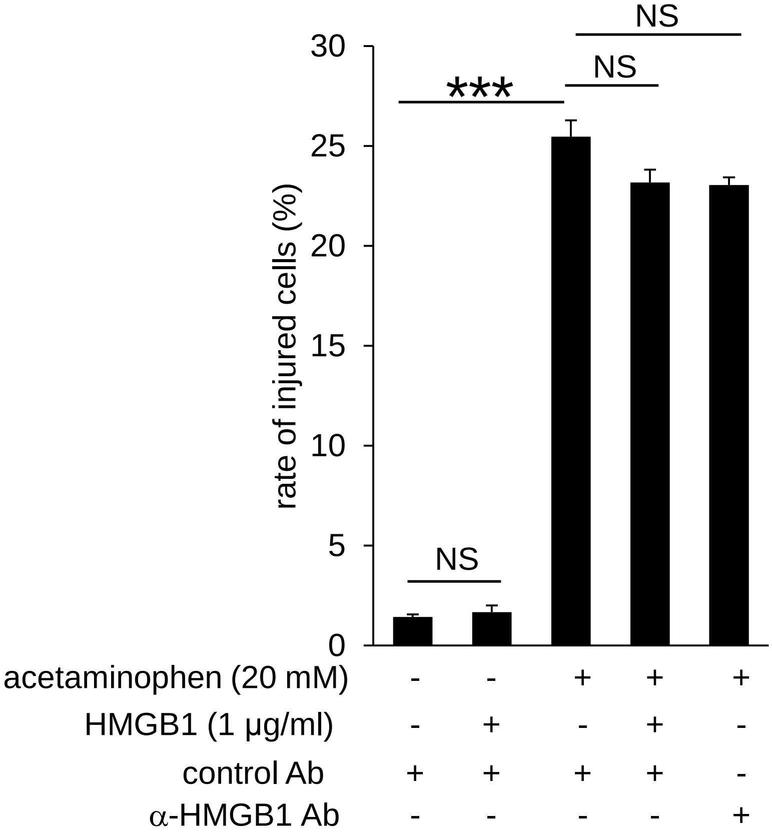

Supplemental Figure. 3 HMGB1 had no effect on the acetaminophen-induced injury of hepatocytes *in vitro*
HepG2 cells were incubated with 20 mM acetaminophen, HMGB1 at 1 μg/ml, and anti-HMGB1 neutralizing antibody at 1.5 μg/ml at 37℃ for 24 h. Then the cells were stained with Hoechst, cell permeable DNA dye (blue) and propidium iodide, cell impermeable DNA dye (red), the number of cells were visually counted. (A) The rate of injured cells (magenta) were calculated. Data are presented as mean ± SEM (n = 3). NS; not significant, ***; *P* < 0.001.
(B) Representative data of microscopy images. Scale bar; 200 μm.

B

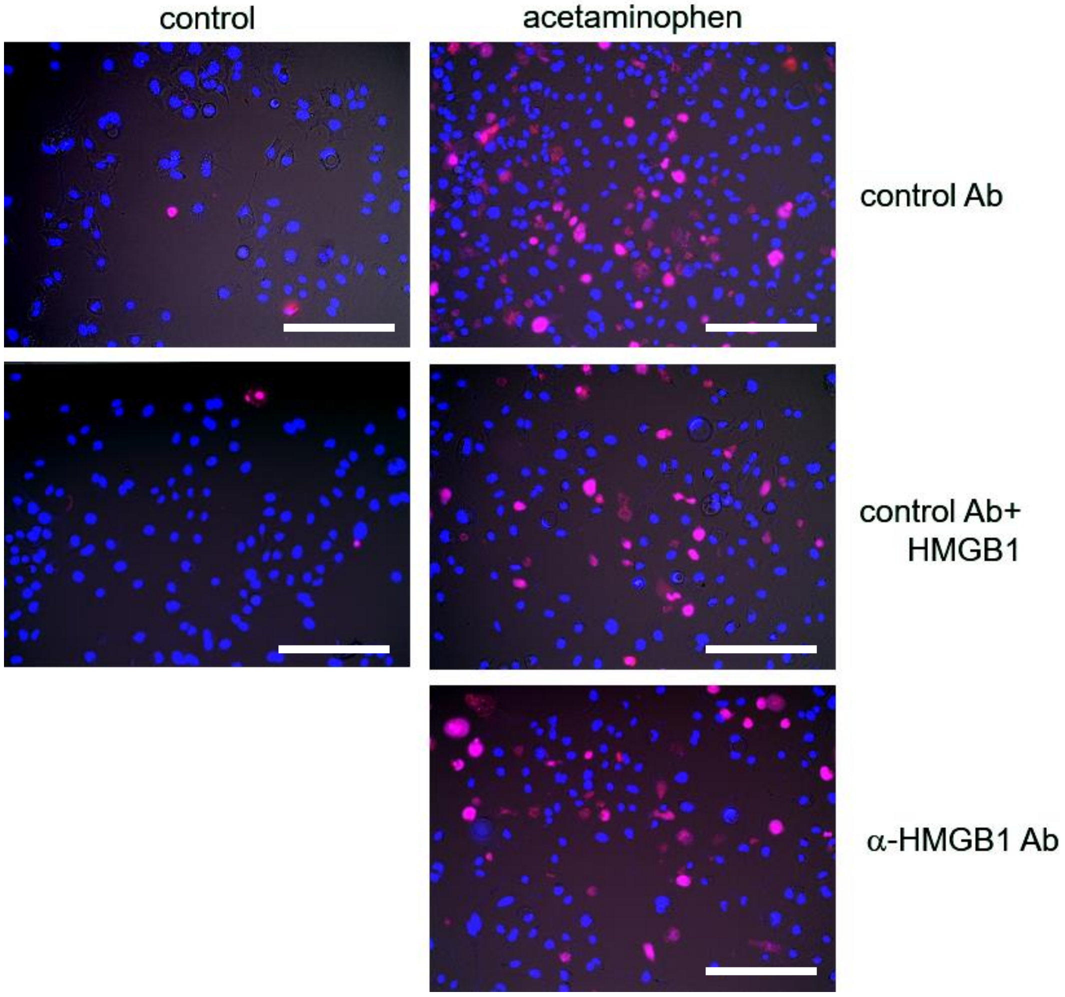

Supplemental Figure. 4

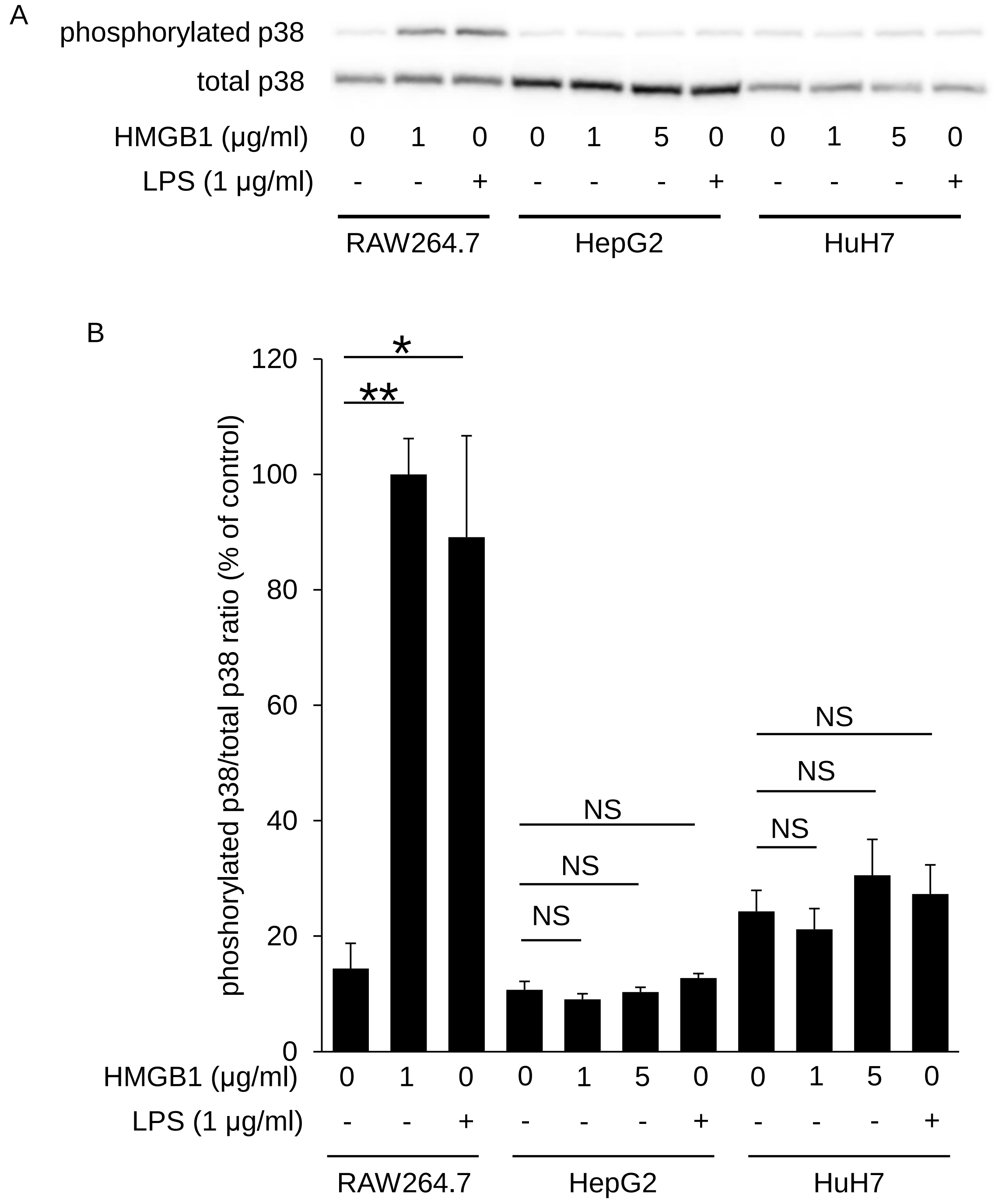

Supplemental Figure. 4: HMGB1 induced no inflammatory response in cultured hepatocytes

RAW264.7, HepG2, and Huh7 cells were incubated with 0-5 μg/ml of HMGB1 or 0-1 μg/ml of LPS. Cell lysates were subsequently analyzed by immunoblotting for phosphorylated and total p38. (A) Typical photos of immunoblots. Data shown are representative of four independent experiments with similar results. (B) Ratios of phosphorylated/total p38 was calculated from densitometry analysis. Results were analyzed as the percentage of control group (no HMGB1 and LPS group in each cell line). Data are presented as mean ± SEM (n = 3). NS; not significant, *; *P*<0.05, and **; *P* < 0.01 vs. control group.
